# Cost-effectiveness of targeted screening for active pulmonary tuberculosis among asylum-seekers: A modelling study with screening data from a German federal state (2002-2015)

**DOI:** 10.1371/journal.pone.0241852

**Published:** 2020-11-05

**Authors:** Katharina Wahedi, Louise Biddle, Kayvan Bozorgmehr

**Affiliations:** 1 Department of General Practice and Health Services Research, University Hospital Heidelberg, Heidelberg, Germany; 2 Department of Population Medicine and Health Services Research, School of Public Health, Bielefeld University, Bielefeld, Germany; The University of Georgia, UNITED STATES

## Abstract

Screening asylum-seekers for active pulmonary tuberculosis is common practice among many European countries with low incidence of tuberculosis. The reported yields vary substantially, partly due to the heterogeneous and dynamic nature of asylum-seeking populations. Rather than screening all new arrivals (indiscriminate screening), a few countries apply targeted screening based on incidence of tuberculosis in asylum-seekers’ country of origin. However, evaluations of its cost-effectiveness have been scarce. The aim of this modelling study was to assess whether the introduction of a screening threshold based on the tuberculosis incidence in the country of origin is sensible from an economic perspective. To this end, we compare the current, indiscriminate screening policy for pulmonary tuberculosis in Germany with a *hypothetical* targeted screening programme using several potential screening thresholds based on WHO-reported incidence of tuberculosis in countries of origin. Screening data is taken from a large German federal state over 14 years (2002–2015). Incremental cost-effectiveness is measured as cost per case found and cost per case prevented. Our analysis shows that incremental cost-effectiveness ratios (ICERs) of screening asylum-seekers from countries with an incidence of 50 to 250/100,000 range between 15,000€ and 17,000€ per additional case found when compared to lower thresholds. The ICER for screening asylum-seekers from countries with an incidence <50/100,000 is 112,000€ per additional case found. Costs per case prevented show a similar increase in costs. The high cost per case found and per case prevented at the <50/100,000 threshold scenario suggests this threshold to be a sensible cut-off for targeted screening. Acknowledging that no screening measure can find all cases of tuberculosis, and that reactivation of latent infections makes up a large proportion of foreign-born cases, targeting asylum-seekers from countries with an incidence above 50/100,000 is likely to be a more reasonable screening measure for the prevention and control of tuberculosis than indiscriminate screening measures.

## 1 Introduction

Tuberculosis is an airborne infectious disease that ranks among the top ten causes of death worldwide [1]. Its global distribution has been heavily influenced by both socioeconomic development and migration: European migrants introduced the currently dominant strain of disease to populations in Africa, Asia and the Americas, where it had been formerly unknown [2]. During industrialization, tuberculosis was the leading cause of death in most European countries, but it has steadily declined since due to improved living conditions, treatment, public health control measures and mass radiography screening, reaching incidences <10/100,000 in many countries [3]. In many low- and middle-income countries in Africa, Asia and the Americas, however, tuberculosis remains highly endemic [1]. Indiscriminate screening measures have been abandoned in low-endemic countries and screening is recommended only for defined high-risk groups, for example people living with HIV, migrants from high-endemic settings, subpopulations with poor access to health care or prisoners [4].

Due to increased migration flows, the number of foreign-born tuberculosis patients has come to outnumber native-born cases in many low-incidence countries, causing renewed interest in screening programmes for asylum-seekers and refugees. These screening programmes exist in many countries with high-income and high net-migration flows [5], but they vary widely in many aspects: stage of disease (active/latent disease), the group of migrants included (all migrants/long-term migrants/asylum-seekers/only from selected high-endemic countries), the timing of the screening (pre/upon/post-arrival), the legal character (compulsory/voluntary), and the follow-up to or the consequence of the screening result (entrance only permitted after successful therapy/surveillance/no follow up) [6, 7].

Despite substantial descriptive literature on the differences of existing screening programmes, evidence on the effectiveness—beyond the reporting of yields—and the cost-effectiveness of the different screening policies for active tuberculosis is scarce [8]. The few economic evaluations that do exist have applied different endpoints: some have calculated costs per case *detected* (excluding treatment) [9, 10], others have evaluated costs per case *prevented* under the premise that latent disease is detected and treated [11, 12]. One study compared costs per case actively and passively found (including treatment) [13]. Overall, most studies treat migrants as a homogenous group or study only one specific subgroup (e.g. students from India and China [9]).

Newly arriving asylum-seekers demonstrate varying risks for tuberculosis based on individual factors (sex, age, contact status, chronic diseases [14]), clinical symptoms [15], and pre- and peri-migration factors (country of origin [16, 17], migration route [18]). The underlying risk of the screened population has a substantial impact on the number needed to screen (NNS), positive predictive values of the applied diagnostic tests [19] and, consequently, effectiveness and cost-effectiveness of the screening programmes. A well-designed, targeted screening programme would therefore take all these factors into consideration.

A recent modelling study from Germany showed that introducing a screening threshold based on the incidence of tuberculosis in asylum seeker’s country of origin (e.g. 50 or 100 per 100,000) could increase the effectiveness of the screening programme considerably without major losses in sensitivity [16]. A few countries, such as the Netherlands, Sweden and the United Kingdom screen for active tuberculosis based on incidence of tuberculosis in the country of origin [7]. However, the effect of different thresholds on cost-effectiveness of such a procedure has not been evaluated in detail. In this modelling study, our aim was to assess whether the introduction of a screening threshold based on the incidence in the country of origin is sensible from an economic perspective. To this end, we compare the current, indiscriminate screening policy for pulmonary tuberculosis in Germany with a *hypothetical* targeted screening programme using several potential screening thresholds. We weigh up expected benefits (cases detected and cases prevented) with expected costs for each strategy, calculating incremental cost effectiveness with respect to costs per case detected and costs per case prevented.

## 2 Methods

### 2.1 Study population

According to the German Asylum Law (§62 AsylG) and Infection Protection Law (IfSG §36) all arriving asylum-seekers must undergo a mandatory health examination. It includes a symptom-based interview and a chest x-ray for asylum-seekers ≥16 years, excluding pregnant women. Responsible actors may vary between states and/or regions. For the study population, we used data on yield of screening, including country of origin and final diagnosis, from the screening programme in one of the largest German federal states (Baden-Württemberg; 2002–2015). During this time period, the regional public health agency was responsible for overseeing and implementing the screening process.

For these 14 years, data from 119,037 asylum-seekers (≥18 years) were available, among which 98 cases of pulmonary tuberculosis had been detected by screening [20]. We decided to limit our population to those countries for which ≥5 cases were identified during screening *or* ≥ 5000 asylum seekers were screened *and* country-level estimates of tuberculosis were available in the WHO data base [21]. The reason for this, as reported elsewhere in details [16], was that reasonable statistical accuracy in estimates of country-stratified yields based on regression models can only be expected with a minimum of numbers screened or minimum of cases found. The sample fulfilling these criteria, as reported in the underlying reference paper [16], was a population of 84,505 asylum seekers from eleven countries and 73 prevalent cases of tuberculosis. For a detailed reporting of socio-demographics, country-specific number of individuals screened, cases found per country, yields and NNS we refer to already published material [16] The screening protocol consisted of a chest X-ray for all individuals aged 16 years or above, and further diagnostics based on the results of the initial test (for details of the screening protocol see [16, 20]).

### 2.2 Model assumptions

**Fig 1:**
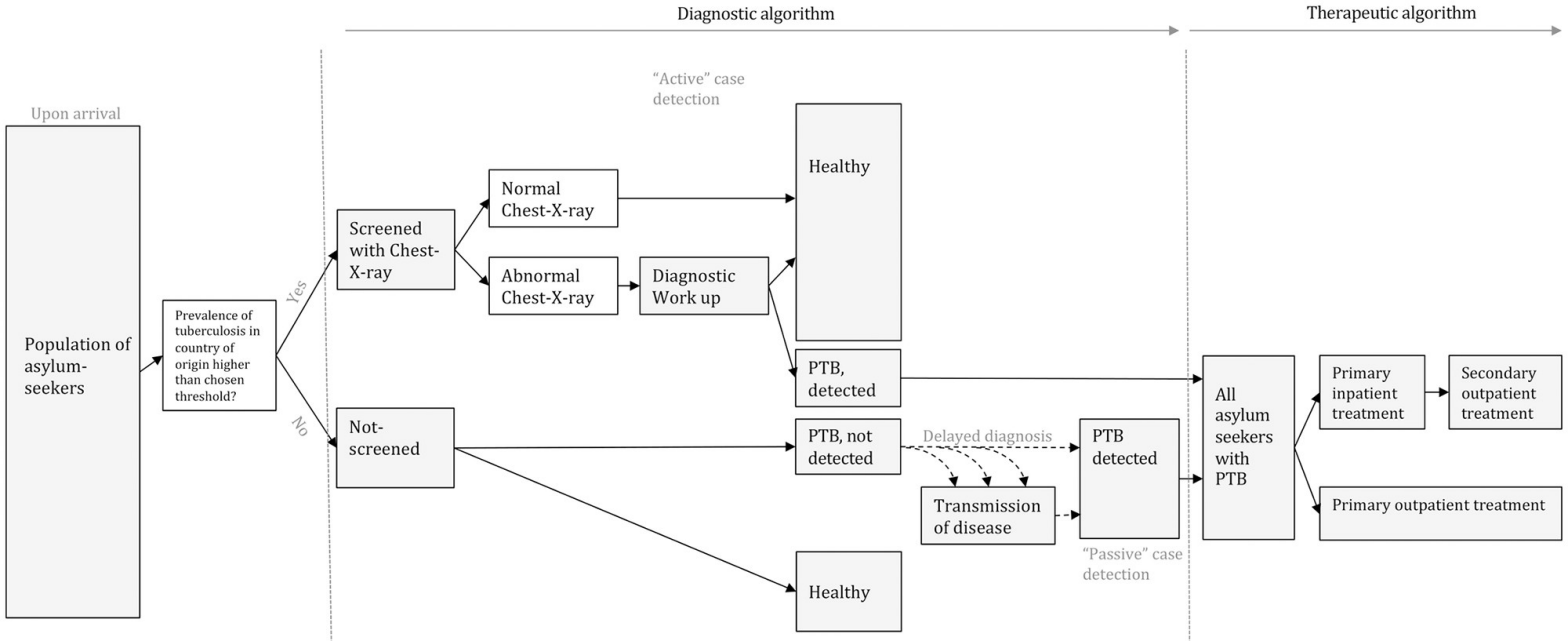
Decision tree of the screening process. PTB = Pulmonary tuberculosis. Asylum-seekers either undergo screening for tuberculosis by chest X-ray or are not screened based on a range of hypothetically chosen thresholds derived from the TB incidence per 100,000 population in their country of origin. Chest X-rays suggestive of tuberculosis are followed up by a clinical examination, microscopy and culture. If an asylum-seeker is diagnosed with pulmonary tuberculosis, they then start treatment. All asylum-seekers with prevalent tuberculosis who are not detected through screening are assumed to become symptomatic and be detected “passively”.

We chose to base our model targeted screening on incidence of tuberculosis in the respective country of origin. Because of the lack of data on the screening process parameters, these were modelled using health economic methods. To this end, we designed a decision tree (see Fig 1) that allocates the asylum-seekers from this population either to the “screening” arm or the “no screening” arm, depending on a range of hypothetically chosen thresholds derived from the TB incidence per 100,000 population in their country of origin (as reported by WHO [21], period averaged incidence in supplements). Screening is performed by chest x-ray, and results suggestive of tuberculosis are followed up by a clinical examination, microscopy and culture of sputum. If an asylum-seeker is diagnosed with pulmonary tuberculosis, they then begin treatment (either outpatient or hospitalized followed by secondary outpatient treatment, see Fig 1). We further assumed that all asylum-seekers with prevalent tuberculosis who are not detected through screening will eventually become symptomatic and be detected “passively”, that is to say, develop symptoms and seek health care. Cases found through screening, in contrast, are referred to as “actively” found cases. Cases detected through active case finding were considered to cause fewer secondary cases by having a shorter infectious period, as explained in more detail below. This difference in secondary cases were considered ‘cases prevented’ through active case finding.

### 2.3 Cost-effectiveness analysis

We performed a cost-effectiveness analysis using costs per case of pulmonary tuberculosis detected through screening and costs per case of pulmonary tuberculosis prevented as primary endpoints. We compared the current practice (no incidence-based threshold for all aged 16 years and above), with hypothetical thresholds at incidence steps of 50/100,000 (50; 100; 150; 200; 250/100,000 population) and a “do nothing” scenario (no screening). This resulted in seven hypothetical alternative screening programmes. As recommended by Drummond *et al*. [19], incremental cost-effectiveness ratios (ICER) were calculated moving from the least costly (no screening) to the most effective and most costly alternative (all screened). To calculate the ICER, each threshold was compared, in turn, with the previous, less costly/ effective threshold. For each scenario comparison, the ICER was defined as dividing additional costs by additional cases detected and additional cases prevented. No scenario had been excluded as extendedly dominated.

Comparing the indiscriminate screening to screening asylum-seekers from countries with a WHO-reported incidence of >50/100,000 was used as base case scenario for sensitivity analyses as it entailed a considerably higher ICER than all other scenarios.

### 2.4 Programme costs

Total programme costs consisted of four components: Screening costs, diagnostic work up to confirm or discard positive screening results (physicians’ visit, culture and microscopy), public health measures (source tracing and contact investigations) and treatment costs (medication, in-patient and out-patient costs). For a shortened list of all included parameters and their costs see Table 1, a full list of parameters is provided in the S1 File.

**Table 1:** Intermediate parameters for individual case definitions, calculated based in part on [22].

Resulting costs for work-up, treatment and public health follow-up	Unit costs
*Resulting inpatient and outpatient costs per case definition*	
Costs for hospitalised, culture positive case	5.963,36 €
Costs for non-hospitalised, culture positive case	1.372,59 €
Costs for hospitalised, culture negative case	5.963,36 €
Costs for non-hospitalised, culture negative case	1.220,89 €
MDR, hospitalised	48.439,23 €
MDR, non-hospitalised	36.601,79 €
*Resulting inpatient and outpatient costs per mode of case finding*	
Costs for actively detected case	4.709,52 €
Costs for passively detected case	4.948,05 €
*Resulting public health costs per mode of case finding*	
Public health costs for actively detected case	702,30 €
Public health costs for passively detected case	820,97 €
*Other costs*	
Costs per work up	134,70 €

Unit costs = unit costs based on the statutory insurance scheme.

To estimate treatment costs, we extracted costs for four different case definitions from a costing study for tuberculosis in Germany [22]: (1) culture positive and hospitalized, (2) culture negative and hospitalized, (3) culture positive and outpatient treatment and (4) culture negative and outpatient treatment, updating the unit costs to 2019 [23]. As there are no standard reimbursement schemes and real cost data is not published, we used unit costs from the statutory insurance scheme [9] for all technical and diagnostic measures except public health measures (contact investigations and source tracing), for which real costs data was available [22]. Medication costs were based on standard therapy regimes [24] and German standardized prices [25].

For sensitivity analyses we also included the reimbursement scheme used by private providers [26]. Public health costs include source tracing via chest-x-ray and contact tracing for culture-confirmed cases via TST and Interferon-y-release assay, as calculated in a cost study for Germany [22].

All costs were calculated from a health-care payer perspective. Costs for health expenditure of asylum-seekers in reception centres in Germany are covered by state or regional administrative authorities based on individual arrangements with hospitals, public health units and physicians. The same is true for the realization of obligatory screening measures. All unit costs were provided in Euros from 2019.

### 2.5 Model parameters: Culture-positive and culture-negative cases, hospitalisation rates and transmission

Passively found cases are considered to be more often infectious (smear positive and/or culture positive [27, 28]) due to delayed diagnosis, more frequently require hospitalization [12, 28], have longer periods of infectiousness and therefore are more likely to transmit disease [29]. A large number of cases missed due to a screening threshold may thus *theoretically* create larger costs than indiscriminate screening due to higher number of passive and secondary cases. As this may impact the (cost)effectiveness of screening once a threshold is introduced, we included these considerations in our model: We assumed the proportion of culture positive cases to be 62 vs. 82 percent for actively vs. passively found cases based on data reported for Germany (2002–2014) [27]. The proportion of hospitalised cases (more severe and expensive course of disease) was derived from data collected continually for Germany (2002–2016) through the Robert Koch-Institute (central public health body in Germany) and was set at 73% for actively found and 78% for passively found cases.

For hospitalisation rates differentiated by type of case finding (active/passive) we used the data collected continually for Germany (2001–2018), provided by the Robert Koch-Institute (central public health body in Germany). For the years 2001–2018 11915 cases were detected actively of which 8718 were hospitalised. 53244 cases were detected passively, of which 41613 were hospitalised. This resulted in hospitalisation rates of 0,732 (0,724–0,739) for actively found cases and 0,782 (0,785–0,778) for passively found cases.

Modelling transmission rates for tuberculosis is complex, as transmission dynamics depend on disease prevalence, strain of tuberculosis, total infectious period and culture/smear positivity [30], years lived in the country and intensity of social contacts of the index case [31], among many others. Molecular epidemiological studies have shown that contact investigations may often fail to identify existing epidemiological links, therefore underestimating transmission and overestimating re-activation of disease [32]. Using molecular clustering as indicator for recent transmission, on the other hand, may overestimate transmission rates because it ignores coincidental simultaneous reactivation, especially from populations with low variability and high stability of strain types [33].

We therefore decided to use a static transmission model, using transmission rates as reported from a clustering study in Hamburg, Germany [32] and calculated by the (n-1) method [34]: As 22.36 (19.47–25.26) percent of the cases in Hamburg resulted from recent transmission, we assumed average transmission rates of 0,28 (0,24–0,34) per passively found case (i.e. about 3–4 passively found cases would cause one additional case of tuberculosis). However, these numbers exclude undetected cases or cases caused with long periods of latency through subsequent infection and reactivation. We therefore assumed five infected contacts per case, of which 5–15% are considered to progress to active disease in their lifetime, therefore causing 0.5 further cases on average, resulting in an overall transmission rate 0.78 per case. We also included a significantly higher transmission rate (up to 3 per case) in our sensitivity analyses to evaluate the resulting effect on costs and cost-effectiveness. For actively identified cases, we assumed that they were detected upon arrival by timely screening, therefore causing no further transmission in Germany.

### 2.6 Sensitivity analyses

All sensitivity analyses were conducted for the comparison between indiscriminate screening and a threshold of ≥50/100,000 inhabitants. We conducted multiple deterministic and probabilistic sensitivity analyses to assess the consistency of our results under parameter and structural uncertainty.

*Univariate* deterministic sensitivity analyses were carried out for the transmission rate, the specificity of the chest X-ray, the rates of hospitalization and culture positivity. Whenever provided, we used upper and lower bound values of the 95-percent confidence interval. Alternatively, we approximated logical maximum and minimum values or increased/decreased the base case parameter by 30 percent (as shown in Table 2). *Multivariate* sensitivity analyses were used to model the overall effectiveness of the screening programme (changes in hospitalization, culture positivity and transmission-rate) and to understand the impact of a change in the severity of hospitalized cases (varying hospitalization time and type of diagnose related group DRG). Further multivariate analyses included a change of reimbursement scheme (private insurance fees for higher-bound estimates), and extreme (max/min) scenarios for various cost-related parameters.

**Table 2:** Relevant process parameters and selected unit costs and frequencies of technical and diagnostic measures.

MODEL PARAMETERS	Base case	Type of DSA conducted	PSA distribution	Standard error	Reference
PROCESS PARAMETERS					
***Case characteristics***					
% Culture positive of actively found	0,65	Upper/Lower CI	Beta	0,0128	Kuehne *et al*. 2018 [27]
% Culture positive of passively found	0,84	Upper/Lower CI	Beta	0,0019	Kuehne *et al*. 2018 [27]
% Hospitalised of actively found	0,73	Upper/Lower CI	Beta	0,0041	Unpublished Data^e^
% Hospitalised of passively found	0,78	Upper/Lower CI	Beta	0,0018	Unpublished Data^e^
***Screening and public health costs***					
Specificity of chest X-ray	0,89	Upper/Lower CI ^b^	Beta	0,01276	Van’t Haag *et al*. 2014 [19]
Public health costs per case for source tracing	297 €	"+/- 30%"	Uniform ^a^	N/A	Diel *et al*. 2012 [22]
Public health costs per case for contact tracing	624,60 €	"+/- 30%"	Uniform ^a^	N/A	Diel *et al*. 2012 [22]
***Treatment and medication***^**9**^					
Mean duration of hospital stay [days]	13,26	Upper/Lower CI	Gamma	0,22551	InEK 2018 [35]
Mean duration only E76A [days]	38,9	Upper/Lower CI	Gamma	0,49460	InEK 2018 [35]
Fraction Cases E 76A/76B/76C	0,21/0,02/0,77	"+/- 30%" ^c^	Uniform ^a^	N/A	InEK 2018 [35]
Costs per day E76C	296,31	"+/- 30%"	Uniform ^a^	N/A	Diel *et al*. 2012 [35]
Quadruple therapy *(INH*, *RMP*, *EMB+ PZA*) [days]	60	None	N/A	N/A	AWMF Guideline TB [24]
Double therapy *(INH+RMP*) [days]	120	None	N/A	N/A	AWMF Guideline TB [24]
Rifampicin (*RMP)* [cost per day]	2,71 €	None	N/A	N/A	“Rote Liste” 2019 [25]
Isoniazid *(INH)* [cost per day]	0,29 €	None	N/A	N/A	“Rote Liste” 2019 [25]
Ethambutol *(EMB)* [cost per day]	2,40 €	None	N/A	N/A	“Rote Liste” 2019 [25]
Pyrazinamide *(PZA)* [cost per day]	1,65 €	None	N/A	N/A	“Rote Liste” 2019 [25]
***Transmission of disease***					
Active cases caused by one case of infectious TB	0,28+0,5 ^f^	Literature	None ^d^	N/A	Sandgren *et al*. 2014, Diel *et al*. 2002 [32, 34]
**DISEASE PREVALENCE IN STUDY POPULATION [Yields per 1000]**					
Macedonia	0,29	Upper/Lower CI	Beta	0,00022	Bozorgmehr *et al*. 2019 [16]
Syria	0,29	Upper/Lower CI	Beta	0,00014	Bozorgmehr *et al*. 2019 [16]
Cosovo	0,34	Upper/Lower CI	Beta	0,00014	Bozorgmehr *et al*. 2019 [16]
Iraq	0,10	Upper/Lower CI	Beta	0,00012	Bozorgmehr *et al*. 2019 [16]
Russia	1,65	Upper/Lower CI	Beta	0,00076	Bozorgmehr *et al*. 2019 [16]
Eritrea	4,64	Upper/Lower CI	Beta	0,00157	Bozorgmehr *et al*. 2019 [16]
Gambia	2,58	Upper/Lower CI	Beta	0,00061	Bozorgmehr *et al*. 2019 [16]
Afghanistan	0,27	Upper/Lower CI	Beta	0,00020	Bozorgmehr *et al*. 2019 [16]
Georgia	2,36	Upper/Lower CI	Beta	0,00109	Bozorgmehr *et al*. 2019 [16]
Cameroon	2,00	Upper/Lower CI	Beta	0,00092	Bozorgmehr *et al*. 2019 [16]
Pakistan	1,37	Upper/Lower CI	Beta	0,00053	Bozorgmehr *et al*. 2019 [16]
Somalia	6,83	Upper/Lower CI	Beta	0,00263	Bozorgmehr *et al*. 2019 [16]
**UNIT COSTS OF TECHNICAL MEASURES (selected)** ^f^					
First doctor’s visit	13,20 €	Literature	None ^d^	N/A	EBM 2019 [23]
Pneumological consultation	19,98 €	Literature	None ^d^	N/A	EBM 2019 [23]
Physical examination of the thorax	0,00 €	Literature	None ^d^	N/A	EBM 2019 [23]
**FREQUENCY OF TECHNICAL MEASURES (selected)** ^f^					
Case definitions: Culture positive & not hospitalised/ Cult negative & not hospitalised/ Cult positive & hospitalised / Culture negative & hospitalised					
First doctor’s visit (per quarter)	1/1/1/1	Expert opinion	Triangular ^a^	N/A	Diel *et al*. 2012 [22]
Pneumological consultation	1/1/0/0	Expert opinion	Triangular ^a^	N/A	Diel *et al*. 2012 [22]
Physical examination of the thorax	1/1/0/0	Expert opinion	Triangular ^a^	N/A	Diel *et al*. 2012 [22]
Initiation of therapy	1/1/0/0	Expert opinion	Triangular ^a^	N/A	Diel *et al*. 2012 [22]

CI = 95% Confidence Interval N/A = not applicable. DSA = Deterministic sensitivity analysis, PSA: Probabilistic sensitivity analysis. Four case definitions for tuberculosis (and two for multi-drug resistant Tuberculosis (MDR-TB) were created by combining culture positivity and hospitalization. For each, the average frequency of diagnostic and therapeutic measures was assumed using a costing study from Germany [22]. For a comprehensive list of all measures and unit costs, including for MDR-TB see S1 File.

^a^ For uniform and triangular distribution upper and lower values were determined using +/- 30 percent of baseline.

^b^ Specificity of chest-x-ray depends on whether any abnormality or abnormality suggestive of TB is given follow-up; so extreme CI of both values were applied.

^c^ Due to the co-dependency of the three values, the first two were added/subtracted 30% of their value and the third adapted so that the sum equalled one.

^d^ For purchasing schemes and transmission rate separate PSAs were conducted.

^e^ Unpublished data provided by the Robert Koch Institute (central public health body in Germany).

^f^ see S1 File for all parameters.

For the probabilistic sensitivity analysis, the specificity of screening via chest x-ray and yields of tuberculosis were assigned beta-distributions, as they are considered to vary between one and zero. 95% Credible Intervals (CrI) for country-specific yield were obtained from Bayesian Poisson regression models as reported elsewhere [20]. Length of hospital stay was assigned a gamma distribution. For the frequency of technical costs, no estimations about uncertainty were available. We therefore made minimal and maximal assumptions, creating triangular distributions. To account for the structural uncertainty of unit costs for treatment and diagnostics, we conducted two probabilistic scenarios, a lower-bound estimate based on unit costs used by the statutory insurance providers [23] and a higher-bound estimate one based on private providers [26]. Medication costs were considered fixed costs.

## 3. Results

### 3.1 Effect and cost of the screening process

A comparison between indiscriminate screening programme and a potential targeted screening programme with a threshold of ≥50/100,000 were used for the base case analysis. Introducing a threshold at a WHO-reported incidence of tuberculosis in the country of origin of ≥50 per 100,000 resulted in 54,468 asylum-seekers from Macedonia, Syria, Kosovo and Iraq not being screened. Screening of the remaining 30,037 asylum seekers detected 79.5% (n = 58) of all prevalent cases of tuberculosis and missed 20.5% (n = 15) of all cases, which through transmission would have caused (at a transmission rate of 0.78) four additional cases of pulmonary tuberculosis through prolonged periods of infectiousness.

The cost of screening (excluding treatment and public health costs) amounted to 2.7 million Euro for the indiscriminate screening and 987,000€ for the targeted screening (threshold ≥50/100,000). This translates into total annual costs of 193,000€ vs. 70,500€ to the payer, per capita costs of 32 vs. 12€ per newly-arrived asylum seeker or 37,000 vs. 17,000€ per identified case for indiscriminate vs. targeted screening, respectively (see Table 3).

**Table 3:** Effect and cost of the screening process.

Strategy	Indiscriminate screening	Targeted screening with threshold 50/100,000
Asylum seekers screened	84505	30037
Asylum seekers not screened	0	54468
Cases found actively	73	58
Cases missed by screening	0	15
Total costs of screening	2.702.237 €	986.853 €
Annual cost of screening	193.017 €	70.490 €
Costs per identified case	37.017 €	17.015 €
Costs per newly arrived asylum seeker	32 €	12 €

### 3.2 Effect and cost of screening, treatment and public health costs (total programme costs)

Total programme cost (including treatment and public health costs of cases found and cases missed by screening) of the indiscriminate screening programme amounted to 3.45 million Euros, of which 343,000€ (11%) were spent over a time span of 14 years on treatment and follow-up of the detected cases. Cost of the individual components of the screening programme are displayed in Table 3. After introducing a threshold at ≥ 50/100,000, total programme costs (including higher treatment and follow-up costs for cases detected later) were 1.4 Million Euro of which 378,000€ (28%) were treatment and follow-up costs. Costs per case detected were 41,700€ (indiscriminate screening) compared to 21,700€ (targeted screening, see Table 4). Introducing a threshold of ≥ 50/100,000 reduced costs per case found by 54 percent with respect to screening costs and by 48 percent with respect to overall costs.

**Table 4:** Total programme costs, cases found and prevented and resulting incremental cost-effectiveness ratios (ICERs).

Threshold	Indiscriminate testing	50/100,000	150/100,000	200/100,000	250/100,000	No screening
*Cost-effectiveness per prevalent case found*						
Cases found	73	58	53	24	14	0
ICER when compared to next higher threshold	110.050,18 €	15.436,24 €	14.040,22 €	10.980,69 €	10.199,47 €	
*Cost-effectiveness per case prevented*						
Cases prevented through screening	56,9	45,2	41,3	18,7	10,9	0,0
ICER when compared to next higher threshold	141.089,98 €	19.790,05 €	18.000,29 €	14.077,81 €	13.076,24 €	
*Further costs*						
Total programme costs	3.046.031,87 €	1.395.279,10 €	1.318.097,89 €	910.931,38 €	801.124,43 €	658.331,90 €
Total screening costs						
Costs per case found actively	41.726,46 €	21.724,23 €	21.466,66 €	19.543,22 €	19.217,71 €	N/A
Costs per case found passively	N/A	9.018,25 €	9.018,25 €	9.018,25 €	9.018,25 €	9.018,25 €
Number of cases found passively	0	15	20	49	59	73

ICER = Incremental cost-effectiveness ratios. ICERs are calculated from the least costly perspective (no screening) to the most costly perspective (indiscriminate screening) and displayed under more costly alternative (e.g. ICER listed under 250/100,000 results from comparing no screening to screening all asylum-seekers from countries with incidences ≥250/100,000.

A passively found case was calculated to cost 7025€ (see Table 4). In principle, an actively found case incurs fewer costs because of lower hospitalization and resulting transmission. However, factoring in the costs for screening procedures, an actively found case was calculated to cost 41,706€ (indiscriminate screening) and 21,704€ (threshold ≥50/100,000). An actively found case is therefore detected at about six times (indiscriminate screening) and three times (threshold≥50/100,000) the cost of a passively found case. Costs per case prevented in the indiscriminate screening were 148,952€ and 84,003€ after introducing a threshold of 50 per 100,000.

### 3.3 Further screening scenarios

Increasing the threshold to 100/100,000 did not lead to the inclusion of any further countries, as no country incidence was described as between 50–100. The threshold of 100 per 100,000 inhabitants therefore did not entail in any change in cost or benefit. Further increasing the threshold to 150/100,000, 200/100,000 and 250/100 decreased programme costs to 2 million Euro, 1.3 million Euro and 1.2 million Euro, finding 53, 24 and 14 cases of tuberculosis, respectively (see Table 4). Completely abandoning screening and detecting all cases passively was calculated to cost 978,000€ and causing 20 additional cases through transmission. All costs for further threshold scenarios can be found in Table 4.

### 3.4 Incremental cost effectiveness

**Fig 2:**
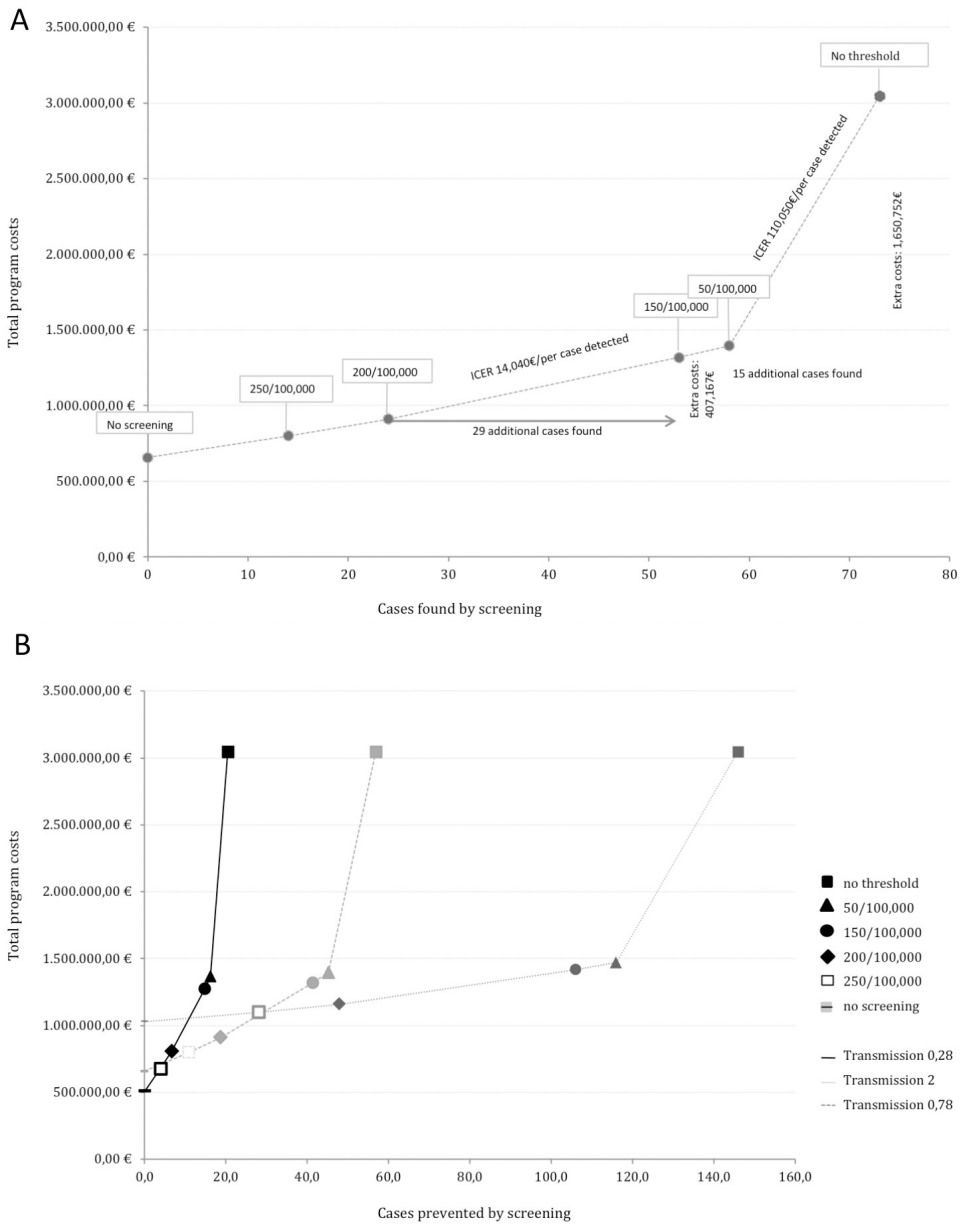
Cost-effectiveness plane of costs and cases *found* (A) and cases *prevented* (B) by screening for six different scenarios. The WHO-reported incidence of tuberculosis/100,000 population in the country of origin was used as threshold to decide whom to include in the screening programme. Six different scenarios were created using different thresholds and are represented by the symbols next to the chosen prevalence. 100/100,000 was not included as distinct plot in the figure, as it did not apply to any of the countries of origin and therefore did not result in a change of ICER or costs. The ICER is calculated based on additional costs per additional cases found by screening (A) or cases prevented (B). It is described by the line connecting the different scenarios; the steeper the line, the higher the ICER. For corresponding ICERs see Table 4. (B) further shows the effect of changing the transmission rate. Independently of the chosen transmission rate (B), the ICER of screening all from countries with a WHO-reported prevalence ≥50 per 100,000 compared with indiscriminate screening is much higher than for any other scenario.

All targeted screening approaches led to ICERs between 15,000€ and 21,000€/per additional case detected through screening when compared to the less inclusive screening strategy, while the indiscriminate screening programme results in an ICER of 165,000€ per additional case detected when compared to the less inclusive alternative (50/100,000), see Table 4 and Fig 2A. For costs per case prevented the difference between ICERs was even more substantial: The ICERs of targeted screening approaches ranged between 43,475 and 62,177€ per additional case prevented, while an indiscriminate screening programme came at 400,000€ per additional case prevented, see Table 4 and Fig 2B.

### 3.5 Deterministic sensitivity analyses

**Fig 3:**
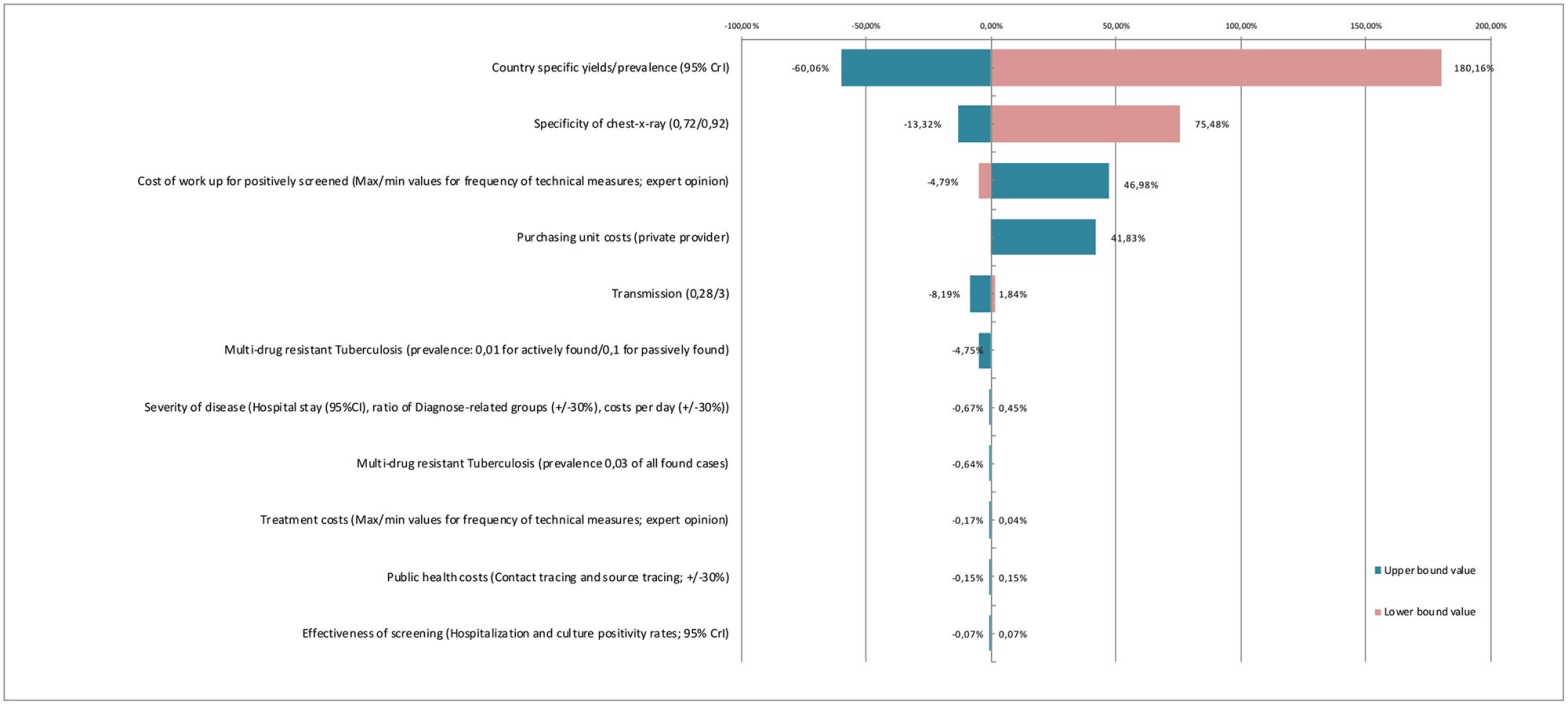
Deterministic sensitivity analysis. Fig 3 shows the results of the deterministic sensitivity analysis for the incremental cost-effectiveness (ICER) of screening only asylum-seekers from countries ≥50/100,000 versus indiscriminate screening. Both univariate (e.g. transmission rate) and multivariate (e.g. changing purchasing costs to unit costs used by private providers) analyses were conducted. The tornado diagram shows the effect of changing these parameters on the resulting ICER. Bars on the right indicate an increase of ICER, bars on the left show a decrease of ICER. Changes resulting from increasing the parameters are represented by green bars while those resulting from a decrease of parameters are shown in red.

The largest changes in ICER resulted from changes in yields (-60%/+180%) followed by the specificity of the chest-X-ray (-13%/+76%), the reimbursement scheme (+42%) and the modelled frequency of diagnostics and technical costs (-5%/47%; see Fig 3). All other parameters had only small effects on the cost-effectiveness of the screening; this includes increasing the transmission rate to two new cases per missed case and considering different rates of multi-drug resistant tuberculosis (MDR).

### 3.6 Probabilistic sensitivity analyses

**Fig 4:**
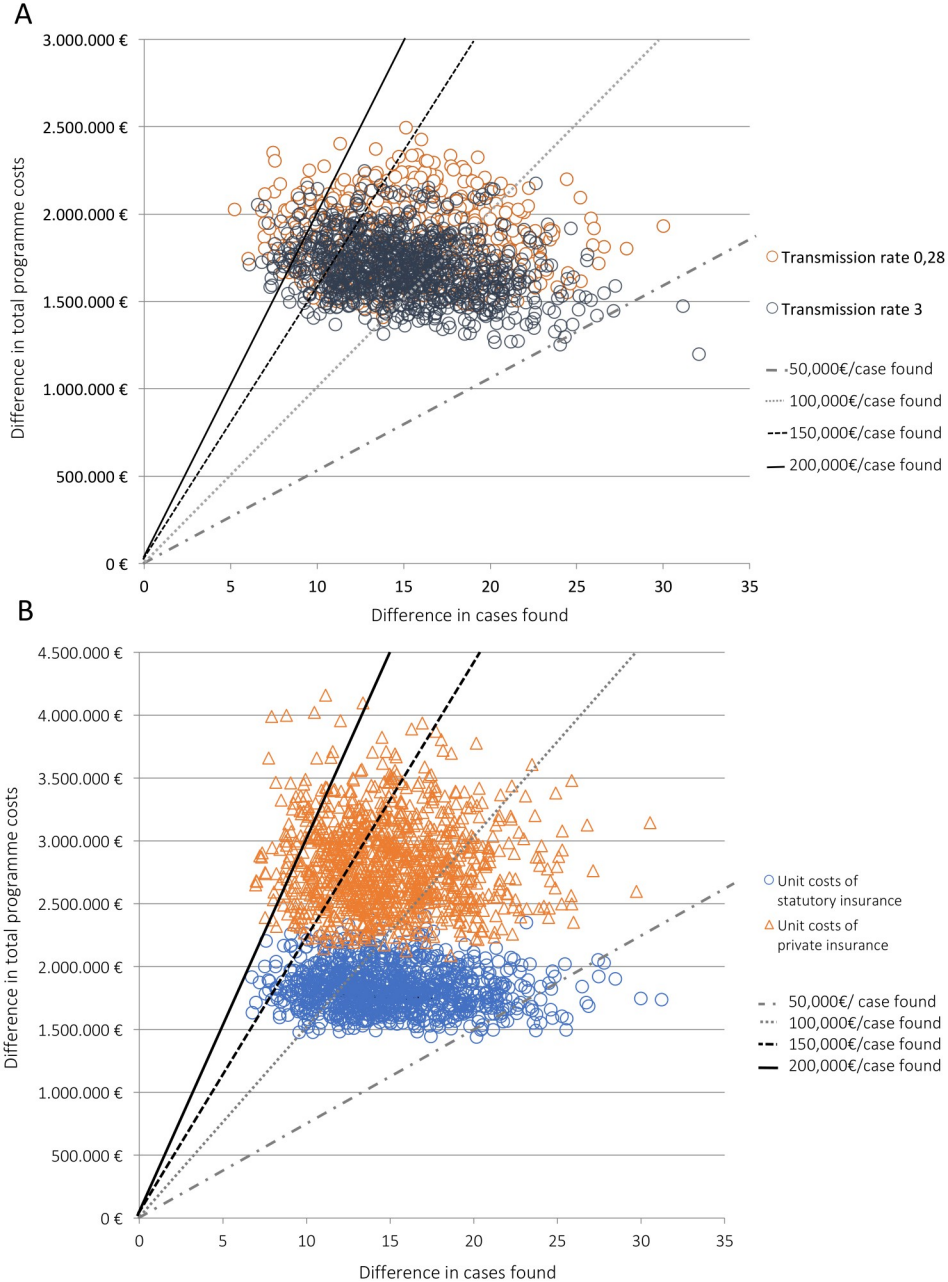
Scatter plot of the joint probability distribution for two different transmission rates and purchasing schemes. A: Probability distribution of 1000 matched pairs (total costs and cases found) for two different transmission rates, 0.28 and 0.3. The mean incremental cost-effectiveness ratio (ICER) was 133,200€ (transmission rate 0.28) and 121,240€ (transmission rate 3) per case found. Four lines mark different “willingness-to-pay”-thresholds, from 50,000€ to 200,000€ per additional case found by screening. B: Probability distribution of 1000 matched pairs (total costs and cases found) for unit costs used by statutory insurance providers unit costs used by private providers, both at a transmission rate of 0.78. Four lines mark different “willingness to pay”-thresholds, from 50,000€ to 200,000€ per additional case found by screening. The mean resulting ICER was 201,509€ (private insurance unit costs) and 130,445€ (statutory insurance unit costs) per additional case found.).

The probability distributions of matched pairs (see Fig 4) show that there is some uncertainty around both costs and cases found, but that the base case represents a conservative estimate, as most points show a less favorable ICER. The probability for cases to be found at costs below 100,000€ per additional case found by screening was low in all scenarios (p = 0.12–0.35).

**Fig 5:**
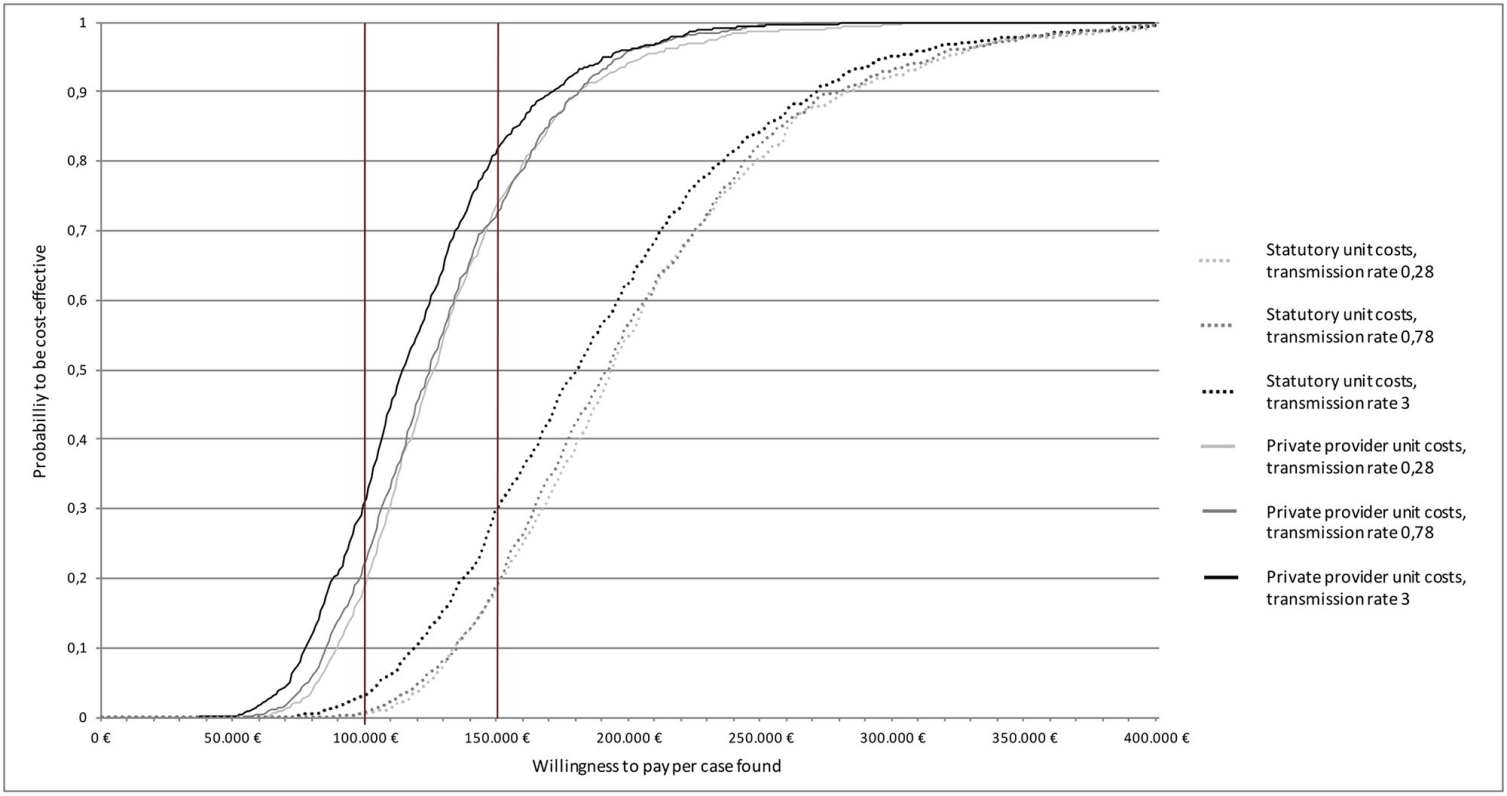
Cost-effectiveness acceptability curve based on transmission rate and reimbursement scheme. Fig 5 shows the cost-effectiveness acceptability curve based on the probabilistic sensitivity analyses for the incremental cost-effectiveness (ICER) of screening only asylum-seekers from countries ≥50/100,000 versus indiscriminate screening. The y-axis depicts the probability of the scenario being below different “willingness-to-pay”- thresholds per case found displayed on the x-axis.

The scenario analysis further shows that different transmission rates did not have a high impact on cost-effectiveness: The probability to find cases below 150,000€ was p = 0.72 (at a transmission rate 0.28) and 0.81 (at a transmission rate 3, see Fig 5). Changing the unit cost to private insurance unit costs, however, considerably lowers the probability of the screening to be cost-effective below 150,000€ per additional case found by screening (from p = 0.72 to 0.19, see Fig 5).

## 4. Discussion

To our knowledge, this is the first study that evaluates in detail how the composition of the screened population impacts cost and cost-effectiveness of screening for active tuberculosis among asylum-seekers. We show that the current, indiscriminate screening programme for pulmonary tuberculosis incurs substantial costs for a comparatively low yield of additional cases when compared to targeted screening programmes based on incidence of tuberculosis in country of origin: Patients from countries with a WHO-reported incidence of tuberculosis <50 per 100,000 are found at far higher costs than those from countries above this threshold. The deterministic and probabilistic sensitivity analyses confirm this result despite uncertainty of the underlying data and show that present calculations are conservative estimates.

Balancing highly targeted against more comprehensive screening approaches is a classic public health dilemma. While indiscriminate screening for tuberculosis comes with relative low harms, we have shown that it does come at a considerable cost, leading to opportunity costs elsewhere. As the challenge for the policy-maker lies in using available resources in the most efficient manner, this requires a careful balance between sensitivity and cost-effectiveness when making decisions about a screening programme. With this in mind, we want to highlight two observations from our analysis, which we believe provide valuable insights for policy makers and public health researchers.

Firstly, in times of higher immigration, indiscriminate *and* timely screening of all arriving asylum-seekers may not feasible due to limited staff and facilities, as has been well documented in Germany [36]. With a high total number of persons to be screened, it may also pose a considerable financial burden on responsible state institutions. In order to maintain sensible screening procedures even in times of high immigration, screening policies need to be adaptive and include strategies to target and prioritize those at highest risk for disease. Our results show that targeting individuals from countries with an incidence rate ≥50 per 100,000 could be a reasonable screening strategy, especially if, for example, implemented within a digital, clinical decision-aid tool that considers tuberculosis incidence in country of origin and further clinical criteria as suggested elsewhere [16]. We further demonstrate that uncertainty in transmission rates does not have a large impact on the cost-effectiveness while the specificity of the screening process and unit costs of diagnostic procedures show considerably higher effects. Due to the high number screened, this means that small changes in specificity may have a large impact on costs. Strategies to improve cost-effectiveness within the existing indiscriminate screening programme could therefore aim to standardize the screening process, for example by defining maximal purchasing costs at the level of public insurance, establishing standardized diagnostic criteria for X-ray-changes suggestive of tuberculosis and defining follow-up work-flows.

Secondly, our analysis challenges the widespread belief that the current screening programme is placed at an acceptable balance between costs and benefits. Paying 110,000€ per case found in asylum seekers from low-incidence countries (<50 per 100,000) is a high price compared to 15–17,000€ per case found in a population with from middle and high incidences (≥50 per 100,000) and also compared to passive case finding at the cost of 9000€. This difference in costs is attributable to the high NNS (>2500) among asylum seekers from low-incidence countries (<50 per 100,000) [16], and raises the normative question of how much money we are willing to spend to find one case of tuberculosis. Even more so, as the benefits of active case finding appear obvious—less transmission of disease, milder course of disease—but evidence to underpin and quantify these effects is sparse and shows only moderate effects [28, 29]. There are several countries that already apply thresholds in the screening of asylum-seekers (e.g. the Netherlands, Sweden and the United Kingdom), however the chosen thresholds are mostly experience-based [7]. Our analysis contributes to existing evidence modelling or evaluating targeted screening approaches [16, 37]. Ideally, a targeted screening would consider information besides country of origin, such as health status, social status and previous contact to the disease, among other factors. Due to a lack of data, we were not able to include these in our model. However, incorporating further risk factors could be easily included in the screening process, as a short interview already forms part of the health screening in Germany. Asylum-seekers may additionally be offered to be screened on a voluntary basis.

Overall, we have demonstrated that considerable benefit could arise from regular monitoring, evaluation and adaptation of established public health programmes such as screening for tuberculosis with respect to the scope, the targeted population and the resulting effectiveness and cost-effectiveness. The Infectious Disease Control Act, which prescribes the screening by chest-X-ray for newly arriving asylum-seekers, was passed in 2001. Even though number of those screened positive are reported on an annual basis, its effectiveness and cost-effectiveness have never been comprehensively evaluated since then, in part due to a lack of denominator data [38]. As a socially constructed, heterogeneous group with a constantly changing composition, asylum-seekers share some health-related risk factors, such as housing, language barriers and restricted access to health care, but differ greatly in others, as has been demonstrated in this analysis with respect to country of origin. As a publicly funded programme, evaluations of effectiveness and cost-effectiveness should be core elements of the programme [39], as these may vary over time [16] and should inform programme design, including screening pathways and the targeted population. Cost-ineffective programmes will otherwise produce high opportunity costs that may fall on alternative screening programmes for tuberculosis, overall public health programmes or the provision of medical health care to asylum-seekers.

### 4.1 Strengths and limitations

We chose a cost-effectiveness analysis over a cost-utility analysis due to the lack of a reliable data basis for answering the question whether screening of asylum seekers at *any* threshold produces individual or public health value that justifies the additional costs, as compared to no screening / passive case finding. Such a study would require valid effectiveness data of TB screening in Germany with respect to morbidity and/or mortality, ideally in forms of effects on quality adjusted life years. Such data do, however, not exist in the German context. We hence refrained from modeling cost-utility based on international studies with data from completely different contexts. The adopted cost-effectiveness approach limits the generalizability of conclusions on the benefits of screening vis-á-vis other (public) health interventions.

As many studies on the cost-effectiveness of screening for tuberculosis use hypothetical cohorts, our study benefits from the use of real screening data of all reported yields over more than a decade in the third-largest state in Germany. Doing so, we do not treat asylum-seekers as a homogenous group while considering a wide range of countries of origin, but provide a comprehensive analysis of the effects of possible thresholds on the cost-effectiveness of a screening programme. We were further able to explore the effect of uncertainty of various parameters through exhaustive sensitivity analyses, both deterministic and probabilistic.

A limitation of many economic analyses for tuberculosis is the complexity of modelling transmission as there is no established method or concept on how to calculate transmission rates for tuberculosis, especially differentiating passively from actively detected cases. As screening programmes are based on this very objective (preventing further transmission by earlier detection) we tried to use the sparse evidence to exist to model this hypothesis. Because there are no trials demonstrating transmission impact of the screening approach and target group that are in focus in this analysis, we had to rely on indirect evidence derived from data of outbreaks and make additional assumptions. However, our sensitivity analysis demonstrated that transmission did not have a large impact on results. Furthermore, we did not have data concerning the prevalence of multi-drug resistant tuberculosis (MDR-TB) or extensively drug resistant tuberculosis, so for the base scenario we did not assume any MDR prevalence. However, we modelled the effect of MDR-cases on the costs and ICER through sensitivity analyses.

For three reasons, we refrained from quantifying the effect of screening for active TB on *quality adjusted life years* (QALYs). First, there is no valid(ated) concept for translating screening for active disease to QALYs [8]. Concepts exist to calculate quality adjusted life years *lost due to* tuberculosis. However, in order to quantify the *effect of screening* for active TB *on QALYs lost or gained* due to tuberculosis we would have to translate the difference in severity and length of disease course of actively vs. passively detected cases to QALYs. For this, no concept has been suggested or studied in detail. Second, there is a lack of an accepted costs/QALY threshold for medical health care provision in Germany. Third, there is structural uncertainty of whether opportunity costs would fall on the health system, all public health programmes or tuberculosis control strategies only. However, in order to quantify money spent and potential benefit of other health interventions, cost-utility analyses will be needed in the future.

Another limitation is that we considered harm with respect to the potential of over-diagnosis only for the first diagnostic step (chest x-ray) with respect to the costs by calculating with low a specificity of the chest x-ray (resulting in a high number of unnecessary and negative cultures/microscopy). The potential of over-diagnosis was, however, not considered for the second step (confirmatory diagnosis, treatment started) as there is no gold standard for “clinical diagnosis” (i.e. culture negative sputum but clinical decision to treat patient as tuberculosis positive). Other negative health outcomes, such as complications by medications, have been considered with respect to their costs.

Future studies on this topic will have to consider the effect of screening for latent disease and treating latent disease. Furthermore, pooled screening data from comparable screening programs collected e.g. in the scope of the E-DETECT program could be used to study cost-effectiveness of different approaches in a wider, international sample to complement the evidence presented here.

## 5 Conclusion

Targeted screening of asylum-seekers from countries with a WHO-reported incidence ≥50 per 100,000 for tuberculosis has substantially lower costs per case found and prevented than indiscriminate screening of all asylum-seekers. The evidence presented in this study challenges the assumption that an indiscriminate screening programme is placed at an acceptable balance between costs and benefits. Understanding asylum-seekers as a heterogeneous population with complex health needs and risks and changing population dynamics will facilitate the design of effective and cost-effective health programmes. Re-designing screening programmes for tuberculosis to target only asylum-seekers from high-incidence countries would be an economically sensible decision based on our analysis. However, in order to further contextualize our results and maximize the use of available resources, tuberculosis programmes also need to be contrasted and compared with other measures improving the health of migrants with respect to their cost-utility.

## Supporting information

S1 File Technical appendix. (DOCX)
