# Supplementary material for: Cost-effectiveness of targeted screening for active pulmonary tuberculosis among asylum-seekers: A modelling study with screening data from a German federal state (2002-2015)
Source: PLoS One. 2020 Nov 5;15(11):e0241852. doi: 10.1371/journal.pone.0241852 (PMC7644037; doi:10.1371/journal.pone.0241852)
Supplement: S1 File — (DOCX) [file pone.0241852.s001.docx]

**S1 File. Technical appendix**

**Further model assumptions**

Due to the imperfect sensitivity of screening via chest-x-ray [2], not all prevalent cases will be found, even in the indiscriminate screening scenario. However, as this applies to all scenarios equally, and no data was available on cases found passively for the study population, sensitivity of the screening procedure was not considered in our calculations.

Case finding was considered to be continuous. Not much is known about the delay in time until cases are found passively, but has been reported to be below one year [3], so no discounting rates were applied.

**Further parameters and costs**

**Sensitivity of chest-x-ray**

Studies of the sensitivity of chest x-rays reported widely differing rates (e.g. 0,79-1 [1]). In our scenario analysis 84,505 asylum seekers were screened and 73 cases of tuberculosis detected. Depending on assumed sensitivity of the screening process, some asylum-seekers with tuberculosis will not have been detected, no matter the screening strategy but rather depending on the assumed prevalence and sensitivity.

These asylum-seekers would therefore need to be included as “passively” detected cases. Nonetheless, our main endpoints stem from comparing scenarios with respect to difference in costs/difference in cases detected through screening and cases prevented. As these costs would have been included in any scenario and does not depend on a chosen threshold, the costs cancel each other out and do not result in any changes of the results. We therefore refrained from making assumptions about the sensitivity of the screening process.

**List of *all* unit cost and frequency of technical measures (addition to table 2)**

| **UNIT COSTS OF TECHNICAL MEASURES ^8^** | | | | | | |
| --- | --- | --- | --- | --- | --- | --- |
|  | Base case | DSA upper | DSA lower | Type of DSA conducted | PSA distribution | Reference |
|  | EBM | GOÄ |  |  |  |  |
| First doctor's visit | 13,20 € | 20,10 € | N/A | Literature | None | [4,5] |
| Pneumological consultation | 19,98 € | 0,00 € | N/A | Literature | None | [4,5] |
| Physical examination of the thorax | 0,00 € | 21,46 € | N/A | Literature | None | [4,5] |
| Initiation of therapy | 0,00 € | 40,23 € | N/A | Literature | None | [4,5] |
| Pneumologist, bronchoscopy | 106,93 € | 120,66 € | N/A | Literature | None | [4,5] |
| Ophthalmological consultation | 13,74 € | 13,41 € | N/A | Literature | None | [4,5] |
| IGRA | 58,00 € | 90,49 € | N/A | Literature | None | [4,5] |
| Transport fee per case | 2,60 € | 0,00 € | N/A | Literature | None | [4,5] |
| HBsAg | 5,50 € | 16,09 € | N/A | Literature | None | [4,5] |
| Microscopy test for Mycobacteria | 5,60 € | 10,73 € | N/A | Literature | None | [4,5] |
| Nucleic acid amplification test | 61,40 € | 20,11 € | N/A | Literature | None | [4,5] |
| Culture test | 34,90 € | 26,81 € | N/A | Literature | None | [4,5] |
| Differentiation of TB bacteria | 28,40 € | 20,11 € | N/A | Literature | None | [4,5] |
| Resistance definition per mycobacteria type | 39,50 € | 20,11 € | N/A | Literature | None | [4,5] |
| Intravenous puncture | 0,00 € | 9,38 € | N/A | Literature | None | [4,5] |
| Creatinine | 0,25 € | 2,68 € | N/A | Literature | None | [4,5] |
| Urea | 0,25 € | 2,68 € | N/A | Literature | None | [4,5] |
| Uric acid | 0,25 € | 2,68 € | N/A | Literature | None | [4,5] |
| Blood count | 1,10 € | 4,03 € | N/A | Literature | None | [4,5] |
| HIV serology | 4,50 € | 20,11 € | N/A | Literature | None | [4,5] |
| Anti-Hbc | 11,10 € | 26,81 € | N/A | Literature | None | [4,5] |
| Bilirubin total | 0,25 € | 2,68 € | N/A | Literature | None | [4,5] |
| GGT | 0,25 € | 2,68 € | N/A | Literature | None | [4,5] |
| GOT | 0,25 € | 2,68 € | N/A | Literature | None | [4,5] |
| GPT | 0,25 € | 2,68 € | N/A | Literature | None | [4,5] |
| Radiograph | 16,45 € | 29,38 € | N/A | Literature | None | [4,5] |
| CT-Scan Thorax | 71,43 € | 209,84 € | N/A | Literature | None | [4,5] |
| **FREQUENCY OF TECHNICAL MEASURES ^9^** | | | | | | |
| Case definitions: Culture positive & not hospitalised/ Cult negative & not hospitalised/ Cult positive & hospitalised / Culture negative & hospitalised/ MDR hospitalised/ MDR not hospitalised (MDR only for base case) | | | | | | |
| First doctor's visit (per quarter) | 1/1/1/1/6/4 | 2/2/2/2 | 1/1/1/1 | Expert opinion | Triangular | [6] |
| Pneumological consultation | 1/1/0/0/1/0 | 3/3/2/2 | 0/0/0/0 | Expert opinion | Triangular | [6] |
| Physical examination of the thorax | 1/1/0/0/1/0 | 3/3/2/2 | 0/0/0/0 | Expert opinion | Triangular | [6] |
| Initiation of therapy | 1/1/0/0/1/1 | 1/1/0/0 | 0/0/0/0 | Expert opinion | Triangular | [6] |
| Pneumologist, bronchoscopy | 1/1/0/0/1/0 | 1/1/0/0 | 0/0/0/0 | Expert opinion | Triangular | [6] |
| Ophthalmological consultation | 1/1/1/1/1/1 | 1/1/1/1 | 1/1/1/1 | Expert opinion | Triangular | [6] |
| IGRA | 0/0/0/0/0/0 | 0/0/0/0 | 0/0/0/0 | Expert opinion | Triangular | [6] |
| Transport fee per case | 3/3/2/2/6/5 | 3/3/3/3 | 3/3/2/2 | Expert opinion | Triangular | [6] |
| HBsAg | 1/1/0/0/1/0 | 1/1/0/0 | 1/1/0/0 | Expert opinion | Triangular | [6] |
| Microscopy test for Mycobacteria | 5/1/1/1/23/18 | 9/3/9/3 | 5/1/1/1 | Expert opinion | Triangular | [6] |
| Nucleic acid amplification test | 1/0/0/0/1/0 | 1/1/0/0 | 1/0/0/0 | Expert opinion | Triangular | [6] |
| Culture test | 4/4/3/3/22/18 | 9/4/9/4 | 4/1/1/1 | Expert opinion | Triangular | [6] |
| Differentiation of TB bacteria | 1/0/0/0/1/0 | 1/0/0/0 | 1/0/0/0 | Expert opinion | Triangular | [6] |
| Resistance definition per mycobacteria type | 1/0/0/0/1/0 | 1/0/0/0 | 1/0/0/0 | Expert opinion | Triangular | [6] |
| Intravenous puncture | 8/7/5/5/22/19 | 10/10/8/8 | 4/4/2/2 | Expert opinion | Triangular | [6] |
| Creatinine | 7/7/5/5/22/19 | 10/10/7/7 | 4/4/2/2 | Expert opinion | Triangular | [6] |
| Urea | 7/7/5/5/22/19 | 10/10/8/8 | 4/4/2/2 | Expert opinion | Triangular | [6] |
| Uric acid | 2/2/0/0/20/19 | 4/4/2/2 | 1/1/0/0 | Expert opinion | Triangular | [6] |
| Blood count | 7/7/5/5/22/19 | 10/10/7/7 | 4/4/2/2 | Expert opinion | Triangular | [6] |
| HIV serology | 1/1/0/0/1/0 | 1/1/0/0 | 1/1/0/0 | Expert opinion | Triangular | [6] |
| Anti-Hbc | 1/1/0/0/1/0 | 1/1/0/0 | 1/1/0/0 | Expert opinion | Triangular | [6] |
| Bilirubin total | 1/1/0/0/1/0 | 10/10/10/10 | 1/1/0/0 | Expert opinion | Triangular | [6] |
| GGT | 8/8/5/5/22/19 | 10/10/7/7 | 4/4/2/2 | Expert opinion | Triangular | [6] |
| GOT | 8/8/5/5/22/19 | 10/10/7/7 | 4/4/2/2 | Expert opinion | Triangular | [6] |
| GPT | 8/8/5/5/22/19 | 10/10/7/7 | 4/4/2/2 | Expert opinion | Triangular | [6] |
| Radiograph | 4/4/2/2/12/9 | 4/4/2/2 | 3/3/2/2 | Expert opinion | Triangular | [6] |
| CT | 0/0/0/0/0/0 | 1/1/0/0 | 0/0/0/0 | Expert opinion | Triangular | [6] |

**Period averaged WHO reported incidence (as reported in [1])**

| **Country** | **Incidence** | **95 % CI (lower)** | **95 % CI (upper)** |
| --- | --- | --- | --- |
| Afghanistan | 189.12 | 172.84 | 206.28 |
| Cameroon | 249.08 | 230.08 | 268.88 |
| Eritrea | 156.48 | 113.84 | 205.64 |
| Gambia | 169.68 | 148.72 | 192.24 |
| Georgia | 205.52 | 196.56 | 215.12 |
| Iraq | 49.16 | 43.16 | 55.64 |
| Kosovo* | 43.7 | 38.79084 | 49.05814 |
| Macedonia | 33.72 | 29.56 | 38.12 |
| Pakistan | 275.44 | 224.6 | 331.4 |
| Russia | 106.16 | 99.24 | 113.36 |
| Somalia | 284.96 | 259.44 | 311.76 |
| Syria | 33.72 | 27.2 | 41.08 |

**References**

[1] WHO | Global Burden of Disease. WHO n.d. http://www.who.int/tb/country/data/download/en/ (accessed October 25, 2019).

[2] van’t Hoog AH, Onozaki I, Lonnroth K. Choosing algorithms for TB screening: a modelling study to compare yield, predictive value and diagnostic burden. BMC Infect Dis 2014;14:532. https://doi.org/10.1186/1471-2334-14-532.

[3] Geisler SS, Helbling P, Zellweger JP, Altpeter ES. Screening for tuberculosis in asylum seekers: comparison of chest radiography with an interview-based system n.d.:8.

[4] Einheitlicher Bewertungsmaßstab - EBM. Bundesgesundheitsministerium n.d. https://www.bundesgesundheitsministerium.de/service/begriffe-von-a-z/e/einheitlicher-bewertungsmassstab-ebm.html (accessed October 8, 2019).

[5] Verband der privaten Krankenversicherung. Gebührenordnung für Ärzte 2008.

[6] Diel R, Rutz S, Castell S, Schaberg T. Tuberculosis: cost of illness in Germany. Eur Respir J 2012;40:143–51. https://doi.org/10.1183/09031936.00204611.
